# Transcriptomics of developing wild sunflower seeds from the extreme ends of a latitudinal gradient differing in seed oil composition

**DOI:** 10.1002/pld3.423

**Published:** 2022-07-22

**Authors:** Max H. Barnhart, Edward V. McAssey, Emily L. Dittmar, John M. Burke

**Affiliations:** ^1^ Department of Plant Biology University of Georgia Athens Georgia USA; ^2^ School of Life Sciences University of Hawai'i at Mānoa Honolulu Hawaii USA

## Abstract

Seed oil composition, an important agronomic trait in cultivated sunflower, varies latitudinally across the native range of its wild progenitor. This pattern is thought to be driven by selection for a higher proportion of saturated fatty acids in southern populations compared with northern populations, likely due to the different temperatures experienced during seed germination. To investigate whether these differences in fatty acid composition between northern and southern populations correspond to transcriptional variation in the expression of genes involved in fatty acid metabolism, we sequenced RNA from developing seeds of sunflowers from Texas, USA, and Saskatchewan, Canada (the extreme ends of sunflower's latitudinal range) grown in a common garden. We found 4,741 genes to be differentially expressed between Texas and Canada, including several genes involved in lipid metabolism. Several differentially expressed lipid metabolism genes also colocalized with known oil quantitative trait loci (QTL). The genes producing stearoyl‐ACP‐desaturases (*SAD*) were of particular interest because of their known role in the conversion of fully saturated into unsaturated fatty acids. Two *SAD* genes were more highly expressed in seeds from Canadian populations, consistent with the observation of increased levels of unsaturated fatty acids in seeds from that region. We also constructed a gene co‐expression network to investigate regional variation in network modules. The results of this analysis revealed regional differentiation for eight of 12 modules but no clear relationship with oil biosynthesis. Overall, the differential expression of *SAD* genes offers a partial explanation for the observed differences in seed oil composition between Texas and Canada, while the expression patterns of other metabolic genes suggest complex regulation of fatty acid production and usage across latitudes.

## 1 INTRODUCTION

Clinal patterns of phenotypic variation are common in nature and often track environmental gradients such as latitude or elevation (De Frenne et al., 2013; Halbritter et al., 2018). Such clines are often attributed to local adaptation, particularly for traits closely associated with life history, due to the importance of matching life history transitions with the local environment (De Frenne et al., 2013; Donohue et al., 2010; Fabian et al., 2015; Stinchcombe et al., 2004). Given the likely importance of traits that covary with environmental clines, there is particular interest in dissecting their genetic basis. Such information can provide insight into the processes that influence the distribution of adaptive genetic variants across species ranges and tolerance to stressful conditions, especially in the context of climate change (Ahuja et al., 2010; Atkins & Travis, 2010).

A particularly intriguing example of clinal trait variation in plants relates to seed oil composition (Linder, 2000; Sanyal et al., 2018). Seed oils are composed of both saturated and unsaturated fatty acids (FAs), the relative proportions of which are thought to influence germination timing and the amount of energy available to seedlings across temperatures (Linder, 2000). While saturated FAs provide more energy than unsaturated FAs, the lower melting point of the unsaturated FAs increases energy availability at cooler temperatures. Notably, a negative relationship between latitude and the proportion of saturated FAs in seeds has been found for several taxa (Linder, 2000; Sanyal et al., 2018). Seed oils from tropical plant species near the equator tend to have significantly greater proportions of saturated FAs than temperate plants in higher latitudes (Linder, 2000; Manos & Stone, 2001). It has thus been suggested that variation in seed oil composition is an adaptation to the temperatures most often experienced during germination (Linder, 2000; Sanyal & Decocq, 2016; Sanyal & Linder, 2013).

**FIGURE 1:**
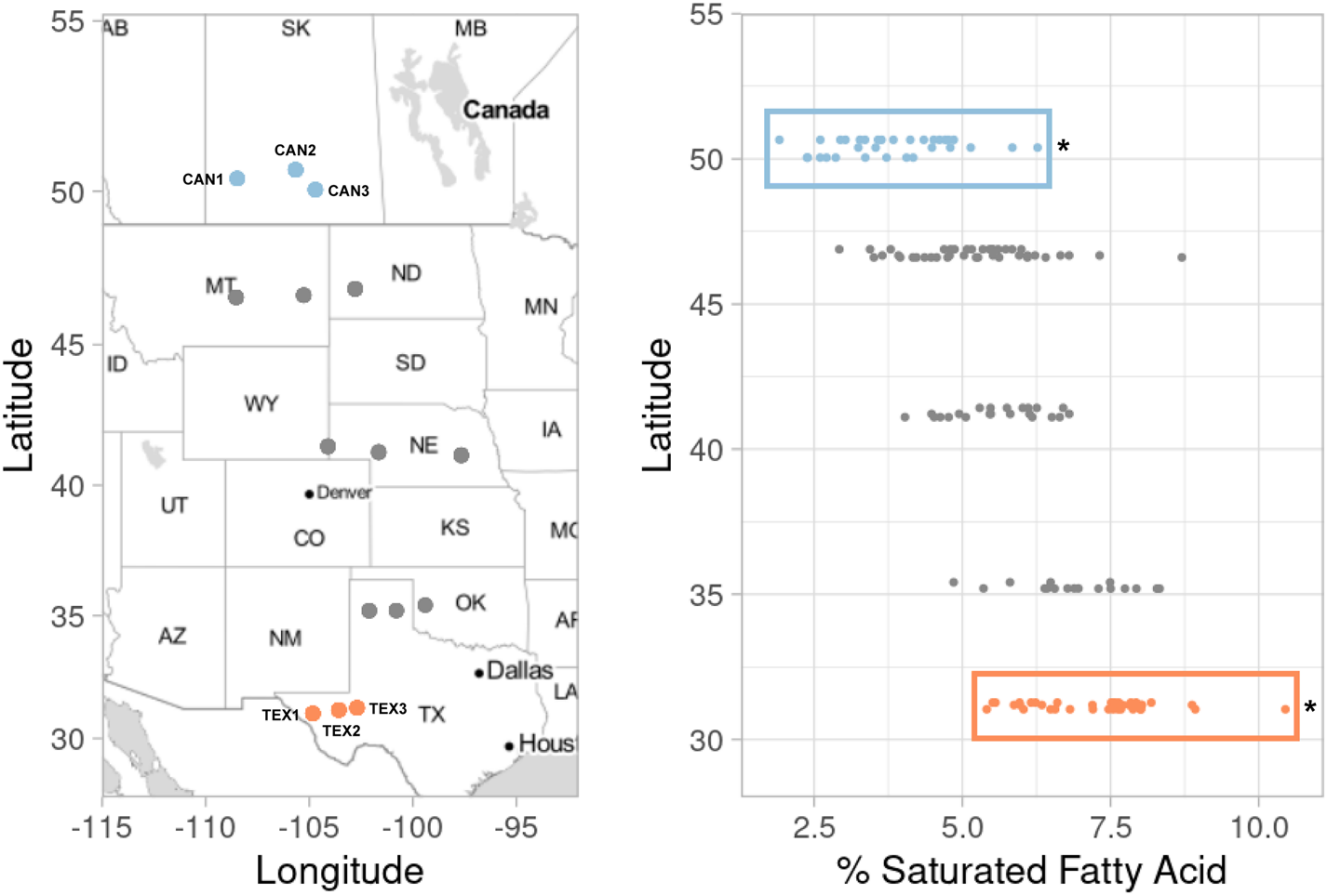
The proportion of saturated fatty acids in seed oils of wild sunflower varies along a latitudinal gradient. (a) Populations of wild sunflowers included in the work of McAssey et al. (2016). Orange and blue dots represent the populations from Texas and Canada that were included in this study. Gray points represent populations studied by McAssey et al. (2016) but not included in this study. (b) Concentration of saturated fatty acids in seed oils from wild sunflower populations across a latitudinal gradient. Samples from Texas are colored orange and grouped within an orange box, while samples from Canada are colored blue and grouped within a blue box. The proportion of saturated fatty acids is significantly higher in seeds from Texas populations than from Canada populations as described in McAssey et al. (2016) and denoted by an asterisk. The seeds used for RNA extraction in this study were produced from the same crosses as those used for oil phenotyping in McAssey et al. (2016).

One of the best‐studied examples of clinal variation in seed oil composition is in wild sunflower (*Helianthus annuus* L.). Across their native range, wild sunflower populations vary latitudinally in the proportion of saturated FAs in their seed oil; that is, southern populations tend to produce seeds with a greater proportion of saturated FAs than northern populations (Figure 1; Linder, 2000; McAssey et al., 2016). Given that this pattern is maintained in a common garden, it seemingly results from genetic differentiation across the species range. Evidence for a trade‐off in germination timing at high and low temperatures between populations from the extreme ends of the gradient has also been observed, consistent with the idea that natural selection on oil composition (due to its effects on germination and/or seedling establishment) is responsible for producing this pattern (Linder, 2000).

In addition to being a model for ecological studies, wild sunflower is the progenitor of cultivated sunflower (also *H. annuus*), which is one of the world's most important oilseed crops. Cultivated sunflower was initially domesticated over 4,000 years ago in what is now the east‐central USA as a source of edible seeds and for cultural purposes (Heiser, 1978; Park & Burke, 2020; Rieseberg & Seiler, 1990) and was later subjected to intense selection for increased seed oil concentration, as well as a shift in FA composition towards greater unsaturated FA content (Fick & Miller, 1997). Sunflower oil is a naturally rich source of oleic and linoleic acids (18:1 and 18:2 respectively, where the numbers represent the length of the carbon chain and the number of double bonds present [i.e., the degree of FA unsaturation]). These two FAs combine to account for approximately 85%–90% of the total seed oil content, with the remainder being largely made up of the fully saturated palmitic (16:0) and stearic (18:0) acids. In recent years, breeding efforts have increasingly focused on the development of high‐oleic lines that produce seed oil composed of ≥ 80% oleic acid, which is valued for its health benefits and stability during storage (Miller et al., 1987).

**FIGURE 2:**
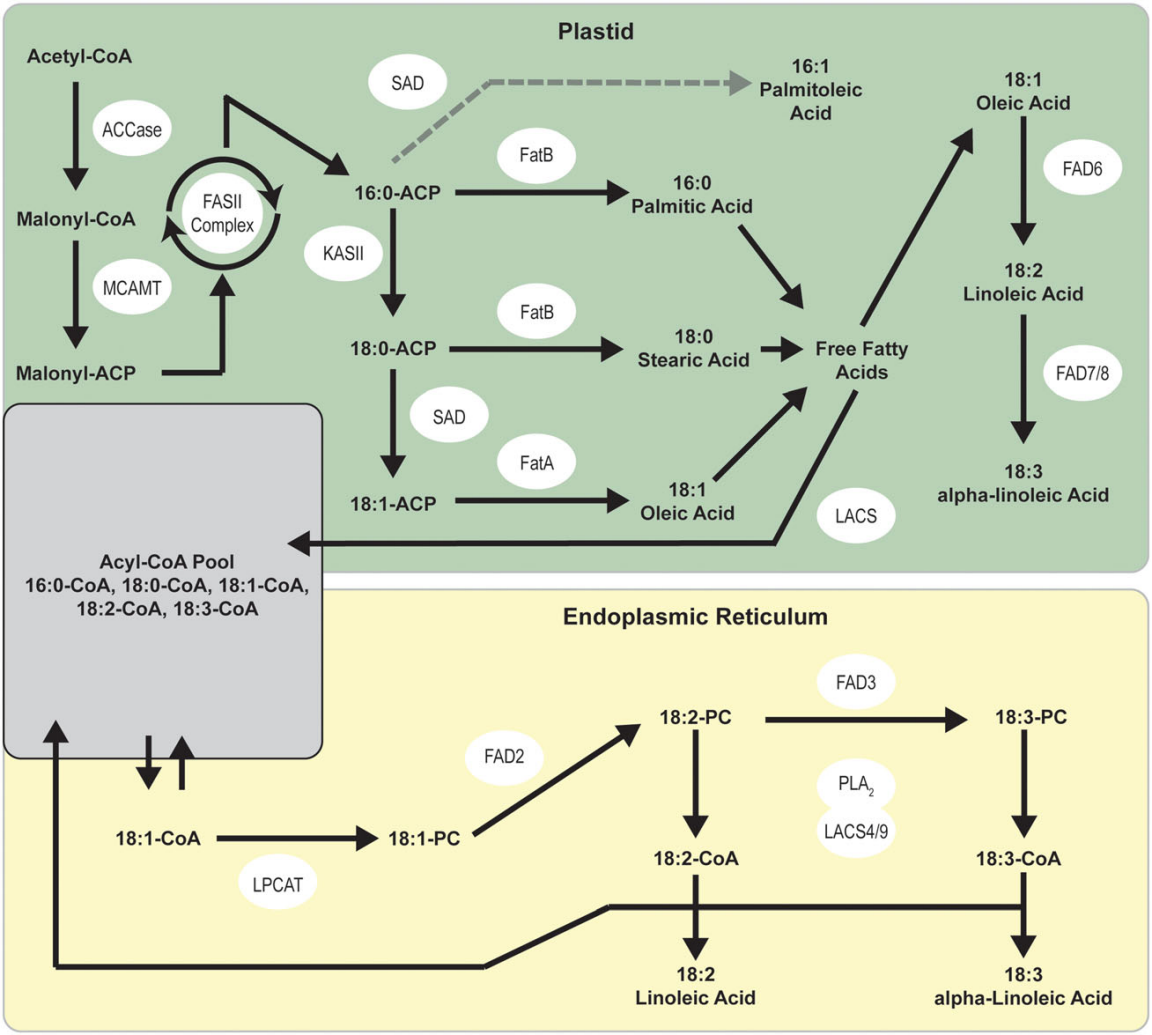
Simplified overview of the plant fatty acid biosynthetic pathway. The first step of fatty acid (FA) biosynthesis in the plastid is the conversion of Acetyl‐coenzyme A (CoA) to malonyl‐CoA by acteyl‐CoA carboxylase (ACCase) which is then further converted to malonyl acyl‐carrier protein (ACP) by malonyl coenzyme A:acyl carrier protein transacylase (MCAMT). From there, fatty acid synthase (FASII complex, a multi‐enzyme complex made up of several fatty acid biosynthesis genes) catalyzes several elongation steps to produce 16:0‐ACP. Ketoacyl‐synthase II (KASII, often considered a component of the FAS complex) further elongates 16:0‐ACP into 18:0‐ACP. SAD then removes the first double bond from these molecules to form a 16:1‐ACP and 18:1‐ACP (SAD exhibits a strong substrate preference for 18:0‐ACP over 16:0‐ACP). FatB and FatA remove the acyl‐carrier protein to form palmitic, stearic, and oleic acid. Long‐chain acyl‐CoA synthetase (LACS) converts free FAs into acyl‐CoAs which are then transferred to the endoplasmic reticulum where lysophosphatidylcholine acyltransferase (LPCAT) removes the CoA and replaces it with phosphatidylcholine (PC). FAD2 then desaturates 18:1‐PC into 18:2‐PC before FAD3 further desaturates that into 18:3‐PC. Those polyunsaturated fatty acid (PUFA)‐PC molecules are then converted into PUFA‐CoA by the combined activity of phospholipase A2 (PLA_2_) and LACS4/9 before finally being converted into linoleic and alpha‐linoleic acid. PUFA‐PC, PUFA‐CoA, and free PUFAs in the ER can all be used to produce other lipid‐based molecules. Oleic acid can also be converted into linoleic and alpha‐linoleic acid in the plastid through the activity of FAD6 and FAD7/8 respectively, but this is not the primary pathway for FA desaturation (Bates et al., 2013; Jung et al., 2019; Ohlrogge et al., 1991).

Due to the ecological and economic importance of seed oil composition and the general importance of FAs in biological systems, the FA metabolic pathway has been well‐characterized (Figure 2; Bates et al., 2013; Jung et al., 2019; Ohlrogge et al., 1991). In plants, palmitic (16:0) and stearic (18:0) acid are the first long‐chain FAs produced during FA synthesis. Stearoyl‐ACP desaturases (SAD) then convert palmitic and stearic acid into monounsaturated palmitoleic (16:1) and oleic (18:1) acid, though the conversion of palmitic acid to palmitoleic acid is relatively minor compared with the conversion of stearic acid to oleic acid. Fatty acid desaturases (FAD) then convert oleic acid into linoleic acid and other polyunsaturated FAs. The role of the enzyme‐producing *SAD* and *FAD* genes in controlling oil composition has been well established in many plant species (Belide et al., 2012; Dar et al., 2017; Fofana et al., 2006; Liu et al., 2002; Rajwade et al., 2014; Thambugala & Cloutier, 2014). In cultivated sunflower, the regulation of these genes is responsible for many commercially important seed oil phenotypes, including the aforementioned high‐oleic acid phenotype, which is conditioned by a mutation resulting in the downregulation of the seed‐specific *FAD2–1* gene (Hongtrakul et al., 1998; Miller et al., 1987; Schuppert et al., 2006). Similarly, increased stearic acid content has been mapped to quantitative trait loci (QTL) containing *SAD* genes (Pérez‐Vich et al., 2004, 2002). While regulation of *SAD* and *FAD* genes underlie these commercially important phenotypes in cultivated sunflower, less is known about how these and related genes contribute to observed variation in seed oil composition in wild populations.

As a follow‐up to observations of latitudinal variation in seed oil composition among wild sunflower populations (McAssey et al., 2016; Figure 1), we sought to characterize transcriptomic variation in developing seeds of plants derived from the southern and northern extremes of the species range. Given what is known about the genetic basis of oil biosynthesis in cultivated sunflower, we asked the following: (1) Which genes are differentially expressed between Texas and Canada and what are their putative biological roles? (2) Are genes related to oil metabolism, particularly members of the *SAD* and *FAD* gene families, differentially expressed between regions? (3) Are differentially expressed genes (DEGs) related to oil metabolism found within known oil QTL? and (4) Are DEGs related to oil metabolism found within co‐expression network modules associated with geographic regions? To answer these questions, we sequenced the transcriptomes of developing seeds from the same crosses of Texan and Canadian sunflower populations that produced the seed for the oil phenotyping performed in McAssey et al. (2016); Figure 1). Overall, several genes related to oil metabolism are differentially expressed across the range of wild sunflower. We found that *SAD* genes specifically are expressed at a significantly higher level in developing seeds from Canadian populations when compared with those from Texan populations, consistent with our expectations given the observed higher levels of desaturation at more northern latitudes. Additionally, many of the DEGs related to oil metabolism were found within oil QTL. Contrary to expectations, however, DEGs related to oil metabolism did not group into a single co‐expression network module associated with region, which suggests that oil metabolism genes do not act in a coordinated fashion to produce the observed seed oil phenotypes. Taken together, our results suggest that the regulation of seed oil metabolism across latitudes is a complex process that is influenced by many different genes.

## 2 MATERIALS AND METHODS

### 2.1 Plant Materials

Seeds used in this study were produced via intrapopulation crosses in a common greenhouse environment during a previous experiment (McAssey et al., 2016). Because both RNA extraction and phenotyping for oil composition are destructive, we were unable to obtain both phenotypic and transcriptomic data from the exact same seed samples. Given this necessary limitation and to make our data as comparable as possible, we extracted RNA from seeds produced by the same crosses and collected at the same time as the seeds used for oil composition analysis in McAssey et al. (2016).

Briefly, seeds from six populations representing the southern and northern ends of the range of wild sunflower (three from Texas, USA, and three from Saskatchewan, Canada; Figure 1 and Table 1) were germinated, allowed to establish, and then transplanted into pots in the greenhouse where they were arranged in a randomized fashion and grown to flowering. Four biological replicates per population were grown and sampled; however, not all samples passed quality control after RNA extraction, as detailed below. Flower heads were bagged once buds began to develop to prevent unintended cross‐pollination. Because wild sunflowers are self‐incompatible, pairs of individuals originating from the same population were manually cross‐pollinated. This involved removing the pollination bags from the two focal plants, collecting pollen from both plants directly into a petri dish, and then using a paintbrush to apply this pollen to receptive florets of the same two plants. Because florets within an inflorescence open over time, pollination was performed over a period of days for each individual. Following pollination, heads were rebagged to prevent contamination and plants were allowed to set seed. Fifteen days after the final pollination, eight developing achenes (i.e., single‐seeded fruits) were collected from the center of each head and pooled together as a single sample for each biological replicate to ensure adequate tissue quantity for RNA extractions. The eight achenes were then placed into 1.5 ml tubes, and frozen in liquid nitrogen. These tubes were then stored at −80°C until RNA extraction.

**TABLE 1:** Accession numbers, location data, and number of maternal plants contributed seed (biological replicates) that passed RNAseq quality thresholds for use in this study

Sample ID	USDA PI#	Location	Latitude	Longitude	Number of biological replicates
TEX1	413,160	Texas, USA	31.03972222	−104.8302778	4
TEX2	664,692	Texas, USA	31.18916667	−103.5780556	1
TEX3	468,476	Texas, USA	31.27277778	−102.6922222	4
CAN1	592,311	Saskatchewan, Canada	50.39361111	−108.4802778	4
CAN2	592,316	Saskatchewan, Canada	50.66	−105.6647222	3
CAN3	592,320	Saskatchewan, Canada	50.0475	−104.7072222	4

### 2.2 RNA extraction and library construction

The eight frozen achenes from each biological replicate were ground with a mortar and pestle using liquid nitrogen and approximately 1 g of polyvinylpolypyrrolidone (PVPP). The ground tissue was then transferred into a tube and placed in liquid nitrogen to keep it frozen. After removing individual samples from the liquid nitrogen for processing, 1 ml of Trizol was added to each tube. The contents were then mixed and allowed to incubate at room temperature for 5 min. Chloroform (300 μl) was then added to each sample, and the tubes were manually shaken and then centrifuged at 12,000 × G for 10 min. The aqueous phase of each sample was then removed via pipetting and transferred to a new tube. After mixing with a 0.53X volume of 100% ethanol, RNA was purified by processing the solution following the Qiagen RNeasy Plant Mini Kit (Valencia, CA, USA) protocol with on‐column DNase digestion.

RNA quality was assessed using an Agilent Bioanalyzer (Santa Clara, CA). Nine samples from Texas and 11 from Canada had RIN values ≥8.5 out of 10 and therefore passed our minimum quality thresholds for use in library construction (Table 1). Libraries were constructed using a Kapa mRNA‐seq kit (Kapa Biosystems, Wilmington, MA). This kit performs size selection using magnetic beads. Libraries were constructed to include a size range of approximately 200–500 bp, and size ranges were checked using a fragment analyzer (Advanced Analytical Technologies, Ankeny, IA). Individual libraries were then quantified via qPCR using Illumina (San Diego, CA) standards, and equimolar amounts of each library were pooled into a single tube and submitted for Illumina NextSeq 500 SE75 sequencing at the Georgia Genomics and Bioinformatics Core (http://dna.uga.edu; Athens, GA). The resulting sequence data are available via the Short Read Archive (SRA) under BioProject PRJNA706177.

The resulting RNAseq data were processed using a custom bioinformatics pipeline (https://github.com/EDitt/Sunflower_RNAseq). Adapter sequences were first trimmed using Trimmomatic v0.36 with default settings (Bolger et al., 2014). Next, because samples were split across four lanes during sequencing, reads from each lane were separately mapped to the XRQv1 sunflower genome assembly (Badouin et al., 2017) using the two‐pass mapping method implemented in STAR v2.6.1c (Dobin et al., 2013). The program RSEM v1.3.1 (Li & Dewey, 2011) was then used to calculate expression levels for each gene in each sample prior to downstream analysis.

### 2.3 Differential expression analyses

DEGs were identified using edgeR v3.24.3 (Robinson et al., 2010). Only genes that were expressed at a threshold of at least one count‐per‐million in two or more samples per region were retained for this analysis. To correct for the difference in library sizes between samples, raw counts were normalized to the trimmed mean of M‐values as recommended by the edgeR user guide. Differential expression analyses are not affected by gene‐length bias as the analyses are only concerned with the relative abundance of individual transcripts between samples, so no gene‐length correction was used during this part of the analysis. The model matrix used to estimate dispersions incorporated source population and region as factors. In addition, we tested for differential expression between population pairs within Texas and Canada, except for TEX2, which only had one sample. The false discovery rate was controlled for by applying a Benjamini–Hochberg correction (Benjamini & Hochberg, 1995) to the set of *P*‐values calculated by edgeR. Due to prior knowledge of the influence of *SAD* and *FAD* genes on FA saturation, we conducted independent tests of differential expression of these genes by comparing the transcripts‐per‐million for each gene between the Texan and Canadian samples using a Wilcoxon signed‐rank test (Wilcoxon, 1945).

Gene ontology (GO) terms for each of the sunflower genes were obtained from the blast2go (Conesa & Götz, 2008) output available on the XRQv1 genome portal (https://www.heliagene.org/). GOseq v1.34.1 (Young et al., 2010) was then used to calculate enrichment of GO terms within the set of differentially expressed genes while accounting for transcript length bias and calculating *P*‐values using Wallenius' noncentral hypergeometric distribution (Wallenius, 1963) after which the false discovery rate was controlled for by applying a Benjamini–Hochberg correction to the raw *P*‐values (Benjamini & Hochberg, 1995).

### 2.4 QTL colocalization

We determined the locations of DEGs relative to the positions of previously mapped QTL related to FA biosynthesis that were compiled in Badouin et al. (2017) from several other previously published works (Ebrahimi et al., 2008; Pérez‐Vich et al., 2016; Premnath et al., 2016). The HanXRQv1 base pair coordinates of QTL from those three studies were compiled in Badouin et al. (2017) and used in this study. The genomic coordinates of oil QTL from Burke et al., 2005 were determined by the following: (1) identifying the simple‐sequence repeat (SSR) markers surrounding each QTL, (2) locating those SSR markers on the consensus genetic map of the sunflower genome (Bowers et al., 2012), (3) identifying the nearest single‐nucleotide polymorphism (SNP) to each of those markers, and (4) locating the target sequences for each SNP probe (Bachlava et al., 2012) on the sunflower XRQv1 genome via blast and determining their base pair coordinates. All genes within the intervals of interest were then extracted and cross‐referenced with our list of DEGs (supporting information Data Set S1).

### 2.5 Co‐expression network analysis

We built a co‐expression network using CemiTool v3.12 (Russo et al., 2018) in R to test for associations between geographic region (i.e., Texas vs. Canada) and individual co‐expression modules. We generated a signed network using Pearson correlation coefficients based on transcripts‐per‐million normalized counts for all genes that passed the minimum expression threshold for the differential expression analysis. Invariant genes were filtered out from module construction using default settings, and modules were then tested for their association with region using gene‐set enrichment analysis as implemented by CemiTool using the package FGSEA (Korotkevich et al., 2021). Hub genes were identified as the top five most connected genes within each module. Co‐expression modules were then tested for enrichment of GO terms using CemiTool's overrepresentation analysis.

### 2.6 Identification of lipid metabolism genes

To determine which genes in the sunflower genome are involved in oil biosynthesis and overall lipid metabolism, we used a FASTA file containing all genes in the sunflower genome (Badouin et al., 2017) as an input for the Mercator4 tool. This tool is included in the MapMan4 software suite and uses a BLAST based approach to compare gene sequences to those of a curated set of reference proteins (Schwacke et al., 2019). This curated set of proteins is organized into several pathways; genes mapping to the lipid metabolism pathway were identified and then cross‐referenced with the results of our differential expression analysis.

## 3 RESULTS AND DISCUSSION

### 3.1 Which genes are differentially expressed between Texas and Canada and what are their putative biological roles?

#### 3.1.1 Transcriptome sequencing and sample clustering

**FIGURE 3:**
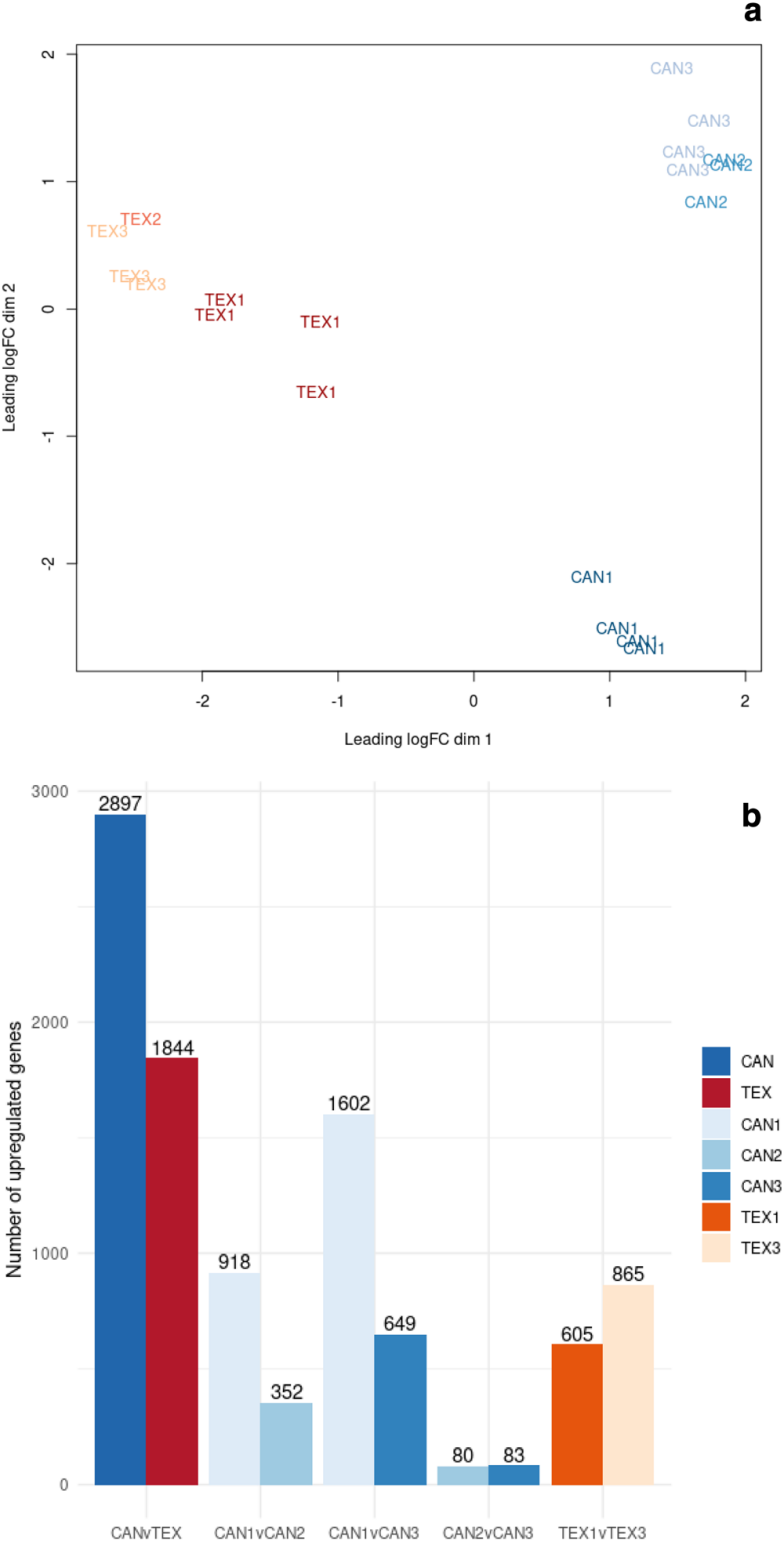
(a) Multidimensional scaling plot illustrating variability between samples/locations. (b) Barplot showing the number of differentially expressed genes between samples from Texas (TEX) and Canada (CAN) as well as the number of differentially expressed genes between individual populations within those regions. Each bar represents the number of upregulated genes in that population relative to the other population in the comparison (i.e., in the CANvTEX comparison, there are 2,897 genes upregulated in CAN relative to TEX and 1,844 genes upregulated in TEX relative to CAN).

Sequencing of the 20 RNA libraries (Table 1) resulted in approximately 559 M total reads, of which ~95% could be assigned to a sample. One sample from Texas with less than 1 M reads was removed prior to the analysis. For the remaining libraries, there was an average of 26.4 M reads per library (ranging from 9.3–85 M reads) with an average of ~18.3 M reads per library mapping to the sunflower genome (supporting information Figure S1). Just over half (29,425 of 58,138; 50.6%) of the genes met the minimum expression threshold of ≥1 count‐per‐million in at least 2 samples and were retained for analysis. Multidimensional scaling (MDS) of samples based upon the expression of all genes passing the minimum expression threshold was used to assess the level of dissimilarity between samples (Figure 3a). Samples from Texas were quite similar to each other, though they did not form a tight cluster. In contrast, samples from Canada were differentiated from those in Texas but formed two distinct clusters with individuals from CAN1 appearing distinct from those of CAN2 and CAN3 (Figure 3a).

A total of 4,741 genes were found to be differentially expressed between the eight samples from Texas and 11 samples from Canada, including 2,897 that were upregulated in Canada and 1,844 that were upregulated in Texas (Figure 3b and supporting information Data Set S2). A smaller number of DEGs were identified in pairwise contrasts between populations within regions (Figure 3b). Consistent with the MDS plot, the CAN2 versus CAN3 comparison had relatively few DEGs, while the CAN1 versus CAN2 and CAN1 versus CAN3 comparisons had much larger sets of DEGs.

As these individuals were grown in a common garden, variation in gene expression is likely due to genetic differences between populations. The clear differences in gene expression between populations from Texas and Canada were expected based on evidence that wild sunflower exhibits north–south divergence in terms of both genetic and phenotypic variation (Blackman et al., 2011; McAssey et al., 2016; Park & Burke, 2020). These patterns likely result, at least in part, from selection for adaptation to the drastic environmental differences between Texas and Canada; such selection might also have contributed to variation in the expression of genes related to environmental adaptation (Akman et al., 2016; Lasky et al., 2014). The CAN1 population is geographically more distant from CAN2 and CAN3 than CAN2 and CAN3 are from each other (Figure 1a) which may result in increased genetic differentiation between CAN1 and the other CAN populations, thereby resulting in a greater number of DEGs.

#### 3.1.2 GO term enrichment within DEGs

Among the 4,741 genes that were differentially expressed in samples from Texas versus Canada, 16 GO terms were found to be significantly enriched (supporting information Data Set S3 and Table 2). Most of the enriched GO terms fell into the molecular function ontology category, with fewer falling into the biological process category, and only a single term being a part of the cellular component category. The enrichment of the single cellular component term, membrane (GO:0016020), could be related to differences in seed oil composition, as the FA composition of membranes is critical for maintaining membrane fluidity at different temperatures (Falcone et al., 2004); however, this is a very high‐level GO term and its relationship to the FA profiles of the seed samples in this analysis is merely speculative. GO terms directly related to FA production, such as lipid metabolism (GO:0006629), omega‐6 FAD activity (GO:0045485), stearoyl‐ACP‐desaturase activity (GO:0102786), and several others, were not found to be enriched within this set of DEGs, even prior to the multiple test correction. We also tested for GO term enrichment among genes upregulated in each region specifically and for each population comparison, but most of these enrichment tests did not yield noteworthy results (supporting information Data Set S3). The lone exception was among genes upregulated in CAN1 versus CAN3 where there was enrichment for genes related to the fatty acid biosynthesis GO term (GO: 0006633). This was somewhat surprising given that the enrichment of this GO term is based upon a comparison between two CAN populations and suggests that overall differences in the expression of genes related to FA biosynthesis between TEX and CAN populations might be driven primarily by the expression of FA biosynthesis genes in CAN1 samples.

**TABLE 2:** GO term enrichment among genes differentially expressed between Texas and Canada

Category	Term	Ontology	*P*‐value	Number of DEGs in category	Total number of genes in category
GO:0000166	Nucleotide binding	Molecular function	5.47E‐06	190	965
GO:0043531	ADP binding	Molecular function	3.32E‐05	48	167
GO:0006952	Defense response	Biological process	7.87E‐05	69	285
GO:0016020	Membrane	Cellular component	7.87E‐05	491	3,010
GO:0016310	Phosphorylation	Biological process	.0001430701155	144	740
GO:0016301	Kinase activity	Molecular function	.0001504724832	120	590
GO:0016740	Transferase activity	Molecular function	.0111670116	136	723
GO:0006468	Protein phosphorylation	Biological process	.01324937678	155	878
GO:0005342	Organic acid transmembrane transporter activity	Molecular function	.02089174127	5	5
GO:0004559	Alpha‐mannosidase activity	Molecular function	.02089174127	8	13
GO:0006013	Mannose metabolic process	Biological process	.02089174127	8	13
GO:0005524	ATP binding	Molecular function	.02089174127	357	2,313
GO:0004672	Protein kinase activity	Molecular function	.02391560147	111	601
GO:0003824	Catalytic activity	Molecular function	.02778733722	64	302
GO:0016787	Hydrolase activity	Molecular function	.02840233826	138	764

Abbreviation: DEG, differentially expressed gene.

### 3.2 Are genes related to oil metabolism, particularly members of the SAD and FAD gene families, differentially expressed between regions?

#### 3.2.1 Expression patterns of SAD and FAD genes

Given their known effects on FA saturation, the expression patterns of *SAD* and *FAD* genes were examined in greater detail to determine if they showed evidence of differential regulation consistent with observed phenotypic differences between regions. Two *SAD* genes and 37 *FAD* genes were expressed in our developing seed samples; however, one *FAD* gene on chromosome 14 was expressed at substantially higher levels than the other 36 *FAD* genes and likely contributes disproportionately to overall FAD enzyme activity.

**FIGURE 4:**
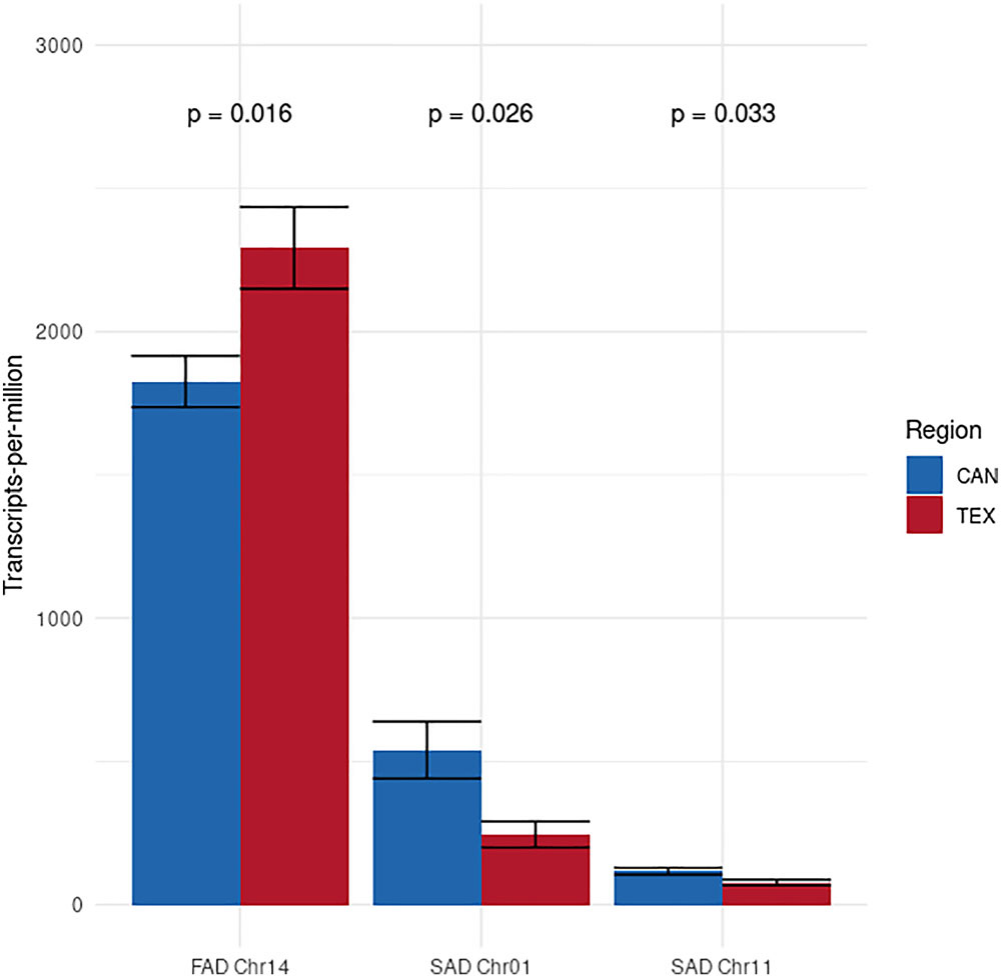
Comparison of expression levels of the two *SAD* genes and the most highly expressed *FAD* gene (*FAD2–1*) among individuals from Texas and Canada. Gene expression is scaled to transcripts‐per‐million. *P*‐values are calculated from a Wilcoxon signed‐rank test. Error bars represent one standard error from the mean.

We conducted independent tests of differential expression on *SAD* and *FAD* genes based upon our prior knowledge of the role of these genes in controlling the conversion of saturated FAs into monounsaturated FAs and, subsequently, into polyunsaturated FAs (Figure 2; *SAD* and *FAD* genes respectively). Using a Wilcoxon rank sum test to compare differences in the TPM of the two *SAD* genes and most highly expressed *FAD* gene between TEX and CAN, we found that there were significant differences in expression for all three genes between regions (Figure 4). Both *SAD* genes were expressed at significantly higher level in CAN versus TEX, while the most highly expressed *FAD* gene was expressed at a significantly higher level in TEX.

Based on the functional role of *SAD* genes reported in other studies (e.g., Cantisán et al., 2000; Pérez‐vich et al., 2002; Pleite et al., 2006; Salas et al., 2004), the increased *SAD* gene expression in developing seeds from Canada compared with Texas is consistent with the observed phenotypic patterns showing higher unsaturated FA content in seeds from this portion of the range (McAssey et al., 2016). Because the transition from fully saturated FAs to monounsaturated FAs is solely mediated by the SAD enzyme (Figure 2), it is thus reasonable to expect that increased expression of *SAD* genes would potentially increase the total number of SAD enzymes produced and therefore convert more saturated FAs into monounsaturated FAs through increased enzymatic activity. While our data cannot definitively show that increased *SAD* gene expression in Canadian sunflowers is the cause of higher seed unsaturated FA content, our results provide strong evidence for future investigation.

#### 3.2.2 Differential expression analysis of other genes in the FA biosynthesis pathway

While the *SAD* and *FAD* genes are primarily responsible for modulating the degree of FA (de)saturation in seed oils (Bates et al., 2013), we also examined the expression of genes within the FA biosynthesis and overall lipid metabolism pathways more broadly. Using the Mercator4 tool, we found 850 genes that mapped to the lipid metabolism pathway curated as part of the MapMan4 software suite. Of those 850 genes, 571 of them passed the minimum expression threshold to be included in our tests for differential expression. Of those 571 expressed genes, 55 of them were differentially expressed between Texas and Canada (supporting information Data Set S4). Aside from several differentially expressed *FAD* genes, few of these DEGs are directly involved in the FA biosynthesis pathway outlined in Figure 2. Many of these DEGs, however, still play a role in long‐chain FA biosynthesis and utilization. For example, several *3‐ketoacyl‐CoA synthase* (*KCS*) genes are more highly expressed in samples from Texas versus Canada. *KCS* genes are involved in the elongation step during the synthesis of very‐long‐chain FAs (Gu et al., 2020; Millar & Kunst, 1997). Drought stress is known to trigger production of these very‐long‐chain FAs, and while they do not affect the overall degree of seed oil saturation, expression of these genes can alter the levels of individual FAs within seed oils (Gu et al., 2020; Guo et al., 2020; Hashim et al., 1993). Members of the ALA‐ALIS flippase complex are also differentially expressed and are involved in the translocation of phospholipids between membrane bilayers (Nintemann et al., 2019). Overall, the fact that several lipid metabolism genes are differentially expressed between regions suggests that there is complex regulation and usage of FAs that go beyond a simple shift in saturated:unsaturated FA ratios and that might be involved in adaptation to the specific environmental conditions experienced in Texas versus Canada.

### 3.3 Are DEGs related to oil metabolism found within oil QTL?

#### 3.3.1 DEG colocalization with oil QTL

We also asked if DEGs were enriched within known sunflower oil QTL from previous mapping studies. We determined the genomic location of DEGs relative to 51 oil QTL from previously published mapping studies in cultivated sunflower and eight oil QTL from a wild × cultivated sunflower cross (Badouin et al., 2017; Burke et al., 2005; Ebrahimi et al., 2008; Pérez‐Vich et al., 2016; Premnath et al., 2016). There were 5,145 expressed genes located within oil QTL, 759 of which were DEGs (supporting information Data Set S5). Among all oil QTL, DEGs were significantly under‐enriched as compared with random expectation (−1.08‐fold enrichment, 828.97 expected DEGs; *P*‐value = .006), while within any individual oil QTL, DEGs were not significantly enriched.

We also asked whether any of the lipid metabolism genes colocalized with any oil QTL. Ten lipid metabolism genes were found within oil QTL; three genes colocalized with the QTL for oleic acid content on chromosome 1 from the wild × cultivated cross, two genes colocalized with the QTL for oleic acid content on chromosome 3 from the wild × cultivated cross, and the remaining five genes individually colocalized with five additional QTL (Table 3). Four of these 10 genes were found to be differentially expressed. One differentially expressed *FAD* gene is found within the oleic acid QTL on chromosome 1 (HanXRQChr01g0011241), and the other differentially expressed *FAD* gene is the most highly expressed *FAD* gene in our data set discussed earlier (HanXRQChr14g0452931). One differentially expressed *KCS* gene is also found within a palmitic acid content QTL on chromosome 8. As noted earlier, *KCS* genes contribute to the production and elongation of very‐long‐chain FAs and are involved in abiotic stress tolerance (Gu et al., 2020; Millar & Kunst, 1997). The final differentially expressed lipid metabolism gene that colocalizes with an oil QTL is a *glycerol‐3‐phosphate acyltransferase* (*ATS1*) found on chromosome 6 (HanXRQChr06g0172791); this gene corresponds to the rate‐limiting enzyme producing lysophosphatidic acid (an important intermediate in the formation of acyl‐lipids) in plants (Chen et al., 2011). These results are consistent with the hypothesis that genes affecting seed oil characteristics vary in their expression within cultivated sunflower and may influence seed oil characteristics in wild sunflower as well.

**TABLE 3:** Oil metabolism genes that colocalize with oil QTL

Gene	Annotation	QTL coordinates (base pair positions)	QTL phenotype (seed oil content)	QTL cross
HanXRQChr01g0003891	phospholipase A1 (PC‐PLA1)	Chr01 125301302‐135301303	Oleic acid	Wild × cultivated
HanXRQChr01g0009721	omega‐3/omega‐6 fatty acid desaturase	Chr01 125301302‐135301303	Oleic acid	Wild × cultivated
**HanXRQChr01g0011241**	omega‐3/omega‐6 fatty acid desaturase	Chr01 125301302‐135301303	Oleic acid	Wild × cultivated
HanXRQChr01g0023271	stearoyl‐ACP desaturase	Chr01 125301302‐135301303	Linoleic, oleic, and stearic acid	Cultivated × cultivated
HanXRQChr03g0092231	dodecenoyl‐CoA isomerase	Chr03 129963181‐168095999	Oleic acid	Wild × cultivated
HanXRQChr03g0092661	3‐ketoacyl‐CoA synthase (KCS)	Chr03 129963181‐168095999	Oleic acid	Wild × cultivated
**HanXRQChr06g0172791**	glycerol‐3‐phosphate acyltransferase (ATS1)	Chr06 6870150‐29342604	Palmitic, oleic, and linoleic acid	Wild × cultivated
**HanXRQChr08g0228321**	3‐ketoacyl‐CoA synthase (KCS)	Chr08 89935992‐99935993	Palmitic acid	Cultivated × cultivated
HanXRQChr09g0237851	ketoacyl‐ACP synthase II (KASII)	Chr09 0‐6460295	Palmitic, stearic, oleic, and linoleic acid	Cultivated × cultivated
HanXRQChr14g0445471	acyl carrier protein (ptACP)	Chr14 123401162‐133401163	Oleic and stearic acid	Cultivated × cultivated
**HanXRQChr14g0452931**	omega‐3/omega‐6 fatty acid desaturase	Chr14 140585231‐158557967	Tocopherol and oleic acid	Cultivated × cultivated
HanXRQChr17g0540371	obtusifoliol 14‐alpha demethylase	Chr17 17637974‐25120357	Palmitic acid	Wild × cultivated

*Note*: QTL coordinates are the range of base pairs on each chromosome across which the QTL can be found. QTL from the cultivated × cultivated crosses were identified by Ebrahimi et al. (2008), Pérez‐Vich et al. (2016), and Premnath et al. (2016) then compiled in Badouin et al. (2017). QTL from the wild × cultivated cross were identified by Burke et al. (2005). Genes in bold were found to be differentially expressed in our analyses.

Abbreviation: QTL, quantitative trait loci.

### 3.4 Are DEGs related to oil metabolism found within co‐expression network modules associated with geographic regions?

#### 3.4.1 Gene co‐expression network analysis

To further examine how gene expression varies between Canada and Texas, we built a gene co‐expression network and tested for module‐specific associations with geographic regions. The variance filtering step reduced the number of genes used for module construction to 3,115. These genes clustered into 12 co‐expression modules, eight of which exhibited significant differences in expression between regions (Table 4 and supporting information Data Set S6). We then conducted a GO term enrichment analysis on each module and found that no modules were significantly enriched for GO terms related to oil metabolism, although module M6 was suggestive of enrichment for genes within the GO category GO:0006633 lipid metabolic process (*P*‐value = .06, supporting information Data Set S6). This module did not, however, exhibit significant region‐specific expression.

**TABLE 4:** Module information from the construction of a gene co‐expression network

Module	# of genes in module	*P*‐value association with Texas	*P*‐value association with Canada	# of genes overlapping with oil QTL	Enrichment score
M1	797	.0006	.0006	108	2.79
M2	530	.0006	.0006	84	2.86
M3	489	.1667	.4444	56	−2.16
M4	242	.7626	.8604	47	−.94
M5	198	.0110	.0114	38	−4.99
M6	177	.9935	.9947	29	.62
M7	169	.0110	.0110	27	−1.42
M8	126	.0076	.0073	25	−4.02
M9	118	.0076	.0073	22	−3.43
M10	106	.6740	.6738	16	.96
M11	85	.0110	.0114	25	1.52
M12	78	.0298	.0425	15	−1.39

Abbreviation: QTL, quantitative trait loci.

The five genes with the highest connectivity within each module were identified as “hub” genes (supporting information Data Set S6). Many of these hub genes were of unknown function while very few were related to lipid metabolism. Module M7 included two GDSL esterase lipase genes and a non‐specific lipid transfer gene as hub genes, while no other module had any hub genes that were seemingly related to lipid metabolism. We then looked for differentially expressed oil metabolism genes within each module, similar to our analysis of oil metabolism genes within QTL. Of the 55 oil‐related DEGs, 11 were found within co‐expression modules (Table 5). In addition, one of the *SAD* genes was also found in a co‐expression module. Only the *FAD* gene HanXRQChr01g0011241 was found both within a co‐expression module and an oil QTL, but this *FAD* gene was lowly expressed among all our samples (between 0.5 and 39.1 TPM, average 14.8 TPM).

**TABLE 5:** Differentially expressed oil metabolism genes found within co‐expression network modules

Module	Gene	Annotation
M1	HanXRQChr05g0138441	Cytosolic NAD‐dependent malate dehydrogenase
M1	HanXRQChr05g0159681	Acyl‐CoA:cholesterol acyltransferase
M1	HanXRQChr06g0174791	steroleosin
M1	HanXRQChr06g0179691	Phospho‐base N‐methyltransferase
M1	HanXRQChr14g0427531	Neutral ceramidase (ncer)
M3	HanXRQChr01g0011241	Omega‐3/omega‐6 fatty acid desaturase
M3	HanXRQChr08g0209891	Sphingosine‐1‐phosphate lyase
M3	HanXRQChr17g0549281	Oleosin
M6	HanXRQChr14g0458271	Dodecenoyl‐CoA isomerase
M6	HanXRQChr01g0023271	Stearoyl‐ACP desaturase
M8	HanXRQChr05g0131921	Alpha chain of ATP‐dependent citrate lyase complex
M8	HanXRQChr06g0172791	Glycerol‐3‐phosphate acyltransferase (ATS1)

While genes related to oil metabolism were found within co‐expression modules, we did not find large groups of oil metabolism genes that were co‐expressed and also varied between regions. This suggests that the variation in seed oil composition between regions is not the result of coordinated gene regulation within a co‐expression module. Instead, variation in seed oil composition is likely the result of several independent processes that do not necessarily act in a coordinated manner.

## 4 CONCLUSIONS

Taken together, our results suggest that the observed differences in seed oil composition between regions likely result from the complex regulation of numerous genes involved in FA metabolism. Evidence suggests that the higher expression of *SAD* genes in Canadian sunflower populations compared with Texan sunflower populations could play a role in altering seed oil composition and contribute to observed patterns of latitudinal variation in oil composition. Furthermore, several other genes related to lipid metabolism are differentially expressed across the range and can be found within oil QTL. While these genes could influence the FA composition of seeds, they could also be involved in other important biological processes related to lipid metabolism. Overall, our results provide insights into the possible role of transcriptomic variation of many genes involved in oil metabolism, and specifically *SAD* and *FAD* genes, in shaping variation in seed oil composition across the range of wild sunflower while also providing avenues for future studies of this ecologically and agronomically important trait.

## CONFLICT OF INTEREST

The authors did not report any conflict of interest.

## AUTHOR CONTRIBUTIONS

MHB analyzed the data and wrote the paper. EVM designed the experiment jointly with JMB, grew the plants, extracted RNA, prepared the sequencing libraries, and contributed to writing the paper. ELD assisted with analyses and contributed to writing the paper. JMB designed the experiment jointly with EVM and contributed to interpretation of analyses and writing of the paper.

## Supporting information


**Data S1.** Supporting Information

**Data S2.** Supporting Information

**Data S3.** Supporting Information

**Data S4.** Supporting Information

**Data S5.** Supporting Information

**Data S6.** Supporting Information

**Figure S1.** Number of reads mapping to the sunflower genome for each sample in our data.
