# Supplementary material for: Transcriptomics of developing wild sunflower seeds from the extreme ends of a latitudinal gradient differing in seed oil composition
Source: Plant Direct. 2022 Jul 22;6(7):e423. doi: 10.1002/pld3.423 (PMC9307388; doi:10.1002/pld3.423)
Supplement: Supplementary file 6 — Data S6. Supporting Information [file PLD3-6-e423-s005.html]

CEMiTool


Code 

- Show All Code
- Hide All Code

# CEMiTool

# Report

## Modules

## Profile Plot

## Gene Set Enrichment Analysis

## Over Representation Analysis

### M1

### M2

### M3

### M4

### M5

### M6

### M7

### M8

### M9

### M10

### M11

### M12

## Interaction Network

### Please add interactions to the CEMiTool object

## Parameters
