# Supplementary material for: Transcriptomics of developing wild sunflower seeds from the extreme ends of a latitudinal gradient differing in seed oil composition
Source: Plant Direct. 2022 Jul 22;6(7):e423. doi: 10.1002/pld3.423 (PMC9307388; doi:10.1002/pld3.423)
Supplement: Supplementary file 7 — Figure S1. Number of reads mapping to the sunflower genome for each sample in our data. [file PLD3-6-e423-s006.pdf]

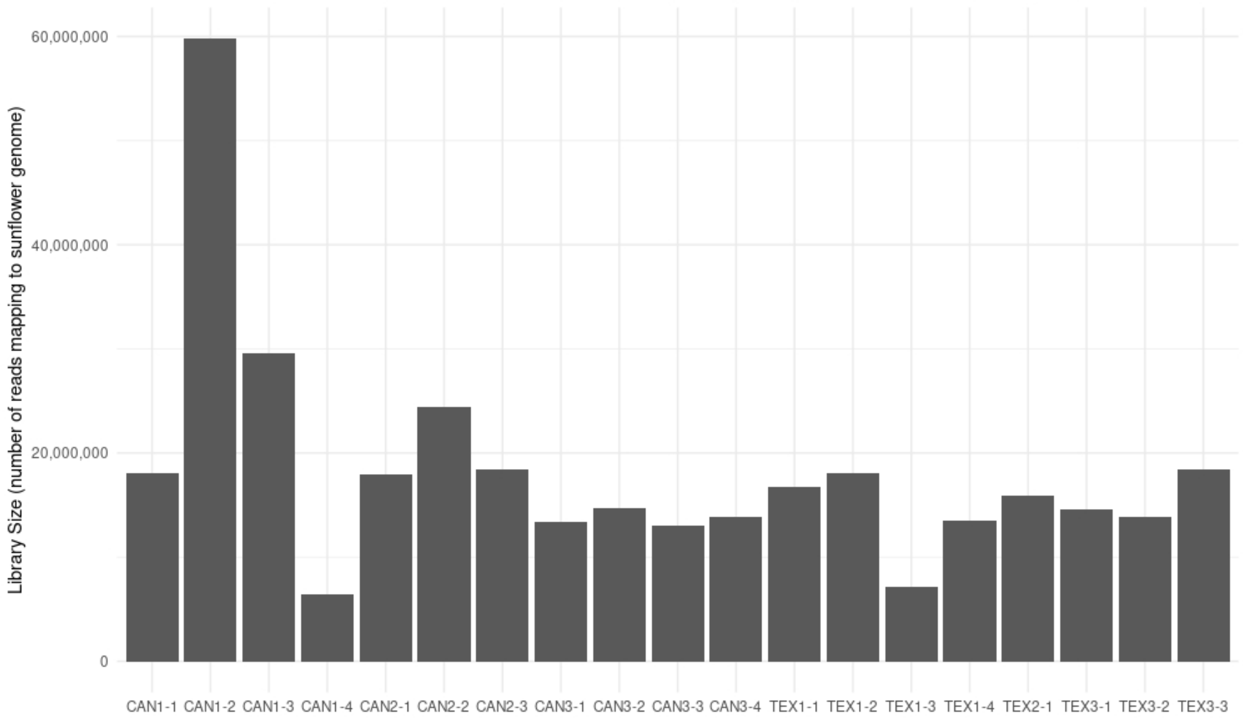

Supplementary Figure 1: Number of reads mapping to the sunflower genome for each sample in our data.
